# Visceral hyperalgesia caused by peptide YY deletion and Y2 receptor antagonism

**DOI:** 10.1038/srep40968

**Published:** 2017-01-20

**Authors:** Ahmed M. Hassan, Piyush Jain, Raphaela Mayerhofer, Esther E. Fröhlich, Aitak Farzi, Florian Reichmann, Herbert Herzog, Peter Holzer

**Affiliations:** 1 Research Unit of Translational Neurogastroenterology, Institute of Experimental and Clinical Pharmacology, Medical University of Graz, Universitätsplatz 4, 8010 Graz, Austria; 2 Neurobiology Research Program, Garvan Institute of Medical Research, 384 Victoria Street, Darlinghurst, Sydney, NSW 2010, Australia

## Abstract

Altered levels of colonic peptide YY (PYY) have been reported in patients suffering from functional and inflammatory bowel disorders. While the involvement of neuropeptide Y (NPY) and Y receptors in the regulation of nociception is well established, the physiological role of PYY in somatic and visceral pain is poorly understood. In this work, the role of PYY in pain sensitivity was evaluated using PYY knockout (PYY^(−/−)^) mice and Y2 receptor ligands. PYY^(−/−)^ mice were more sensitive to somatic thermal pain compared to wild type (WT) mice. Visceral pain was assessed by evaluating pain-related behaviors, mouse grimace scale (MGS) and referred hyperalgesia after intrarectal administration of allyl isothiocyanate (AITC, 1 or 2%) or its vehicle, peanut oil. The pain-related behaviors induced by AITC were significantly exaggerated by PYY deletion, whereas the MGS readout and the referred hyperalgesia were not significantly affected. The Y2 receptor antagonist, BII0246, increased pain-related behaviors in response to intrarectal AITC compared to vehicle treatment while the Y2 receptor agonist, PYY(3–36), did not have a significant effect. These results indicate that endogenous PYY has a hypoalgesic effect on somatic thermal and visceral chemical pain. The effect on visceral pain seems to be mediated by peripheral Y2 receptors.

The gut hormone peptide YY (PYY) is a member of the neuropeptide Y (NPY) family which also includes pancreatic polypeptide. PYY release is regulated by food intake, the parasympathetic nervous system, inflammatory mediators, and other gut hormones including cholecystokinin, vasoactive intestinal polypeptide, gastrin, and glucagon-like peptide-1^1^,^2^,^3^,^4^. PYY is secreted by L-cells of the gastrointestinal tract (GIT) which release it in the form of PYY(1–36). The amino acids tyrosine and proline are cleaved from the N-terminus of the polypeptide by the dipeptidyl peptidase IV (DDP-IV) enzyme to produce PYY(3–36). This changes the pharmacological properties of the peptide. While PYY(1–36) binds to all Y receptor subtypes, PYY(3–36) has a higher affinity to the Y2 subtype^3^. The two peptides have endocrine actions in the GIT where they act as brakes to inhibit gastric emptying, intestinal motility, mouth to anus transit time, and electrolyte secretion in the intestine^1^,^2^.

In addition to regulation of GIT functions and food intake, the NPY system is involved in the regulation of several physiological functions including stress coping, emotional-affective behavior, cognition, neurogenesis, immunity, and nociception^5^. Knockout of Y1 receptors is associated with thermal, chemical, and mechanical somatic hyperalgesia and exaggerated acetic acid- and MgSO_4_ -induced visceral pain^5^,^6^. Electrophysiological studies confirm a role of spinal Y1 and Y2 receptors in controlling postsynaptic currents in pain signaling pathways^7^,^8^. NPY acting through Y1 receptors controls the production of several neuropeptides including substance P and calcitonin gene-related peptide (CGRP), which subserve pronociceptive functions in the CNS^9^. In addition to their effect on spinal pain pathways, Y receptor ligands affect visceral pain mediated by vagal afferent pathways, as c-Fos expression in the nucleus tractus solitarii (NTS) following intragastric acid challenge is enhanced in Y2 and Y4 receptor knockout mice^10^. Furthermore, Y1 receptors are involved in the descending control of pain signaling. For example, NPY injection into the arcuate nucleus reduces somatic pain in rats, an effect that is blocked by Y1 but not Y2 receptor antagonists^11^. In spite of the well-established role of NPY in the regulation of pain sensitivity, the role of the gut hormone PYY in nociception is poorly understood.

Expression of colonic PYY is altered in patients suffering from functional and inflammatory bowel disorders. The density of PYY-containing cells in the colon is lowered in patients with irritable bowel syndrome (IBS)^12^,^13^ and Crohn’s disease (CD)^14^,^15^. Similarly, patients suffering from ulcerative colitis (UC) have lower rectal PYY levels compared to healthy controls^16^. Given that IBS and inflammatory bowel disease (IBD) are associated with pain, we hypothesized that a deficiency in PYY contributes to the hyperalgesia associated with these pathologies.

To examine a possible role of PYY in nociception, we first investigated somatic and visceral pain perception in PYY knockout (PYY^(−/−)^) mice. Somatic sensitivity to thermal pain was tested with the plantar test^17^ while visceral sensitivity to chemical pain was quantified by the pain reactions to intrarectal allyl isothiocyanate (AITC)^18^,^19^. The visceral pain readouts included spontaneous pain-related behaviors, locomotor activity, facial pain expressions, and referred hyperalgesia^18^,^19^,^20^. Concomitantly, the expression of NPY, Y1, and Y2 receptor mRNA in the spinal cord of wild type (WT) and PYY^(−/−)^ mice was assessed. Since PYY(3–36), the main circulatory form of PYY, has a preferential affinity to Y2 receptors^3^, the effects of the Y2 receptor agonist, PYY(3–36), and the Y2 receptor antagonist, BIIE0246, on visceral pain sensitivity were also evaluated.

## Results

### PYY^(−/−)^ mice are hypersensitive to thermal pain

**Figure 1:**
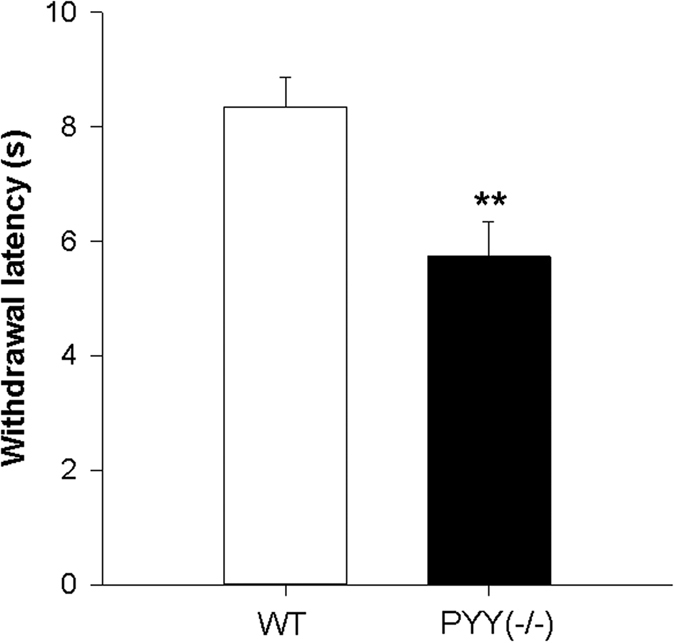
Effect of PYY knockout (PYY^(−/−)^) on withdrawal latency in the plantar test. The data shown are means + SEM, n = 11 per group; **p < 0.01.

Compared to WT mice, PYY^(−/−)^ mice were more sensitive to thermal pain as indicated by a significantly shorter withdrawal latency in the plantar test (t_19.5_ = 3.3; *p* = 0.004) (Fig. 1).

### PYY knockout exaggerates pain-related behaviors induced by intrarectal AITC

**Figure 2:**
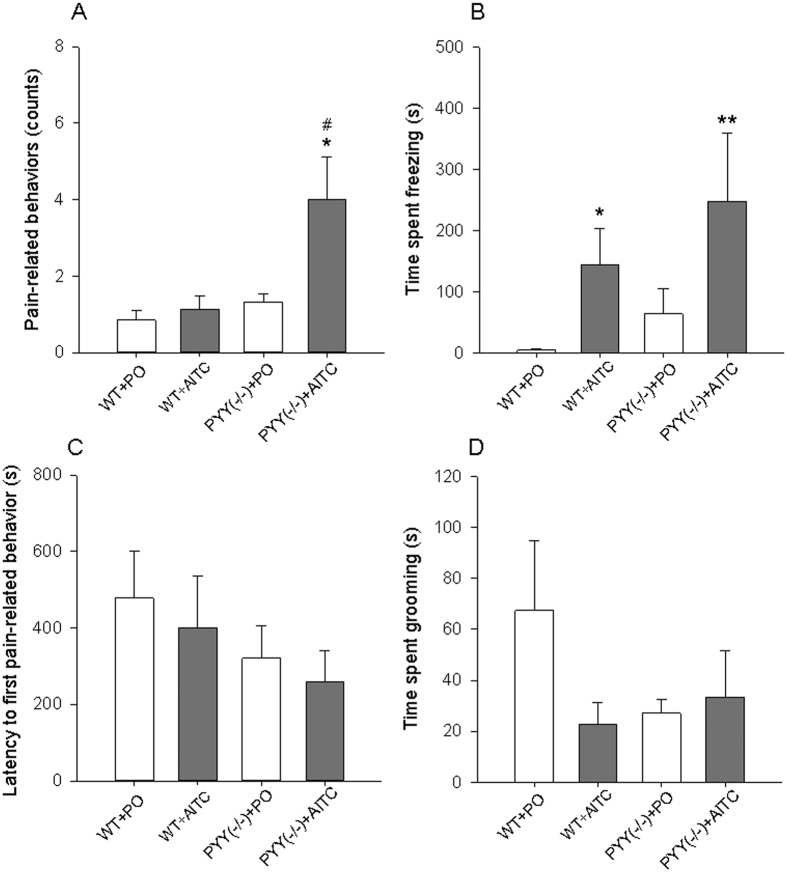
Effect of intrarectally administered AITC (2%, 0.1 ml) and PYY knockout (PYY^(−/−)^) on pain-related behaviors (**A**), time spent freezing (**B**), latency to first pain-related behavior (**C**), and time spent grooming (**D**). The behavioral parameters were evaluated blindly from 15 min videos recorded immediately after intrarectal treatment. One-way ANOVA revealed statistically significant differences among the groups with regard to pain-related behaviors (*p* < 0.05) and the time spent freezing (*p* < 0.01). The data shown are means + SEM, n = 6–8 per group; *p < 0.05, **p < 0.01 compared to WT + PO; ^#^*p* < 0.05 compared to WT + AITC.

Following intrarectal instillation of PO or AITC (2%), one-way ANOVA revealed statistically significant differences in pain-related behaviors (F_(3,24)_ = 4.6; *p = *0.011) and time spent freezing (F_(3,24)_ = 4.6; *p* = 0.003) while the differences in the latency to first pain-related behavior and time spent grooming were not statistically significant (Fig. 2). Post-hoc testing revealed that pain-related behaviors (stretching, squashing, arching and licking of the abdomen) were enhanced in PYY^(−/−)^ mice that received AITC (PYY^(−/−)^ + AITC group) compared to WT mice that received PO (WT + PO group) and WT mice that received AITC (WT + AITC group) (Fig. 2A). The time spent freezing was prolonged in both the WT + AITC and the PYY^(−/−)^ + AITC groups relative to the WT + PO group (Fig. 2B). None of the parameters recorded in the LabMaster system (horizontal activity, vertical activity, traveling distance) during the first 15 min after intrarectal instillation of PO or AITC were significantly different between the experimental groups (Supplement; Figure S1).

### PYY knockout does not alter AITC-induced facial pain expressions

**Figure 3:**
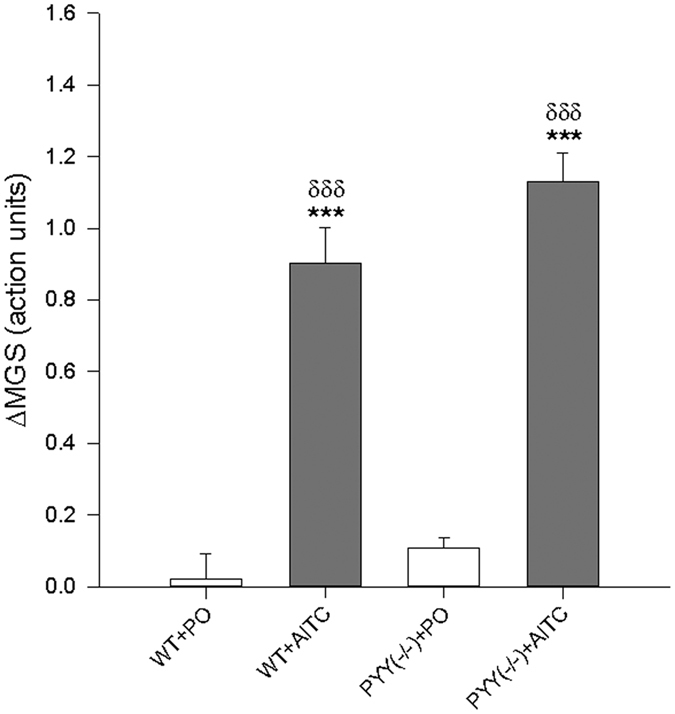
Effect of intrarectally administered AITC (1%, 0.05 ml) and PYY knockout (PYY^(−/−)^) on the mouse grimace scale (ΔMGS) expressed as action units. One-way ANOVA revealed statistically significant differences among the groups (*p* < 0.001). The data shown are means + SEM, n = 6–7 per group; ***p < 0.001 compared to WT + PO; ^*δδδ*^*p* < 0.001 compared to PYY^(−/−)^ + PO.

Following intrarectal instillation of PO or AITC (2%), one-way ANOVA revealed statistically significant differences in ΔMGS among the experimental groups ((F_(3,23)_ = 53.1; *p* < 0.001) (Fig. 3). Post hoc testing revealed that the ΔMGS in the groups which received AITC was significantly higher than in the groups that received PO while other differences between the groups were statistically not significant (Fig. 3).

### PYY knockout does not alter AITC-induced referred hyperalgesia

**Figure 4:**
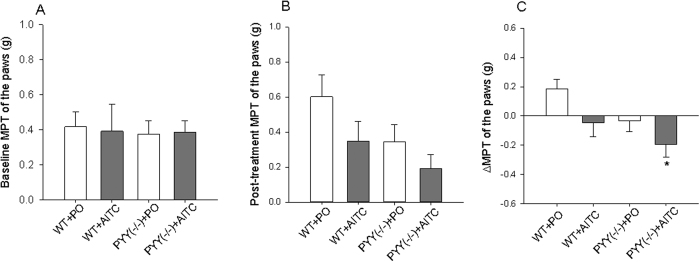
Mechanical pain threshold (MPT) of the plantar surface of the hindpaws in WT and PYY knockout (PYY^(−/−)^) mice before (baseline, **A**) and after (post-treatment, **B**) intrarectal administration of PO or AITC (1%, 0.05 ml) and the difference between the two measurements (ΔMPT, **C**). MPT was assessed with von Frey hairs, and the values represent the average of measurements in both hind paws. With regard to ΔMPT, one-way ANOVA revealed statistically significant differences among the groups (*p* < 0.05). The data shown are means + or − SEM, n = 5–7 per group; *p < 0.05 compared to WT + PO.

The mechanical pain threshold (MPT) on the plantar surface of the hind paws was measured with von Frey filaments in WT and PYY^(−/−)^ mice before and after intrarectal treatment with PO or AITC (1%). The baseline value reflects somatic sensitivity to mechanical pain while the difference between the pre- and post-treatment measurements was taken as index of referred hyperalgesia which accompanies visceral pain. One-way ANOVA failed to disclose any statistically significant differences in MPT among the experimental groups both under baseline conditions and after intrarectal administration of PO and AITC (Fig. 4A,B). A comparison of baseline and post-treatment recordings with the paired *t* test revealed, however, that in the WT + PO group the MPT after instillation of PO was significantly higher than at baseline. In contrast, ΔMPT was significantly different among the experimental groups (F_(3,20)_ = 3.3; *p* = 0.041). Post-hoc testing showed that the ΔMPT was significantly lower in the PYY^(−/−)^ + AITC compared to the WT + PO group but did not significantly differ compared to the WT + AITC group (Fig. 4C).

MPT measurements were also taken on the abdomen. The quantitative MPT and ΔMPT readouts over the abdomen showed a similar but highly variable pattern, and no significant differences could be detected among the experimental groups (Supplement, Figure S2).

### AITC increases spinal NPY and Y1 receptor mRNA expression in PYY^(−/−)^ but not WT mice

**Figure 5:**
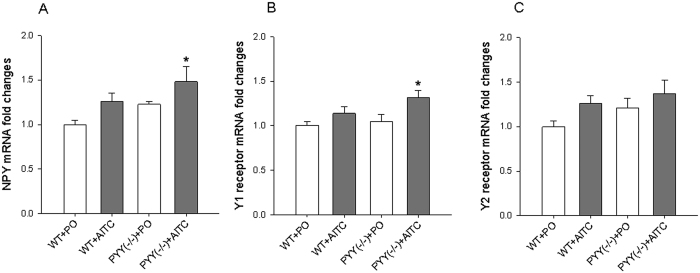
Effect of intrarectal AITC (1%) on the relative expression of NPY (**A**), Y1 receptor (**B**), and Y2 receptor (**C**) mRNA in the lumbosacral spinal cord. Tissue was collected 1 h after intrarectal administration of PO or AITC. Relative mRNA expression was assessed with real time PCR. One-way ANOVA revealed statistically significant differences among the groups with regard to NPY and Y1 receptor but not Y2 receptor mRNA expression. The data shown are means + SEM, n = 5–6 per group; *p < 0.05 compared to WT + PO.

The effect of intrarectal administration of PO and AITC (1%, 0.05 ml) on the expression of NPY as well as Y1 and Y2 receptor mRNA in the lumbosacral spinal cord was examined in WT and PYY^(−/−)^ mice. One-way ANOVA revealed significant differences among the experimental groups with regard to NPY mRNA (F_(3,18)_ = 3.3; *p* = 0.045) (Fig. 5A) and Y1 receptor mRNA (F_(3,18)_ = 3.7; *p* = 0.032) expression (Fig. 5B) while Y2 receptor mRNA expression did not differ to a statistically significant extent (Fig. 5C). Post-hoc testing revealed that NPY mRNA and Y1 mRNA expression in the PYY^(−/−)^ + AITC group was significantly higher than in the WT + PO group (Fig. 5A,B).

### Y2 receptor antagonism increases pain-related behaviors induced by intrarectal AITC

In this experiment, the effects of the Y2 receptor agonist PYY(3–36) (0.2 mg/kg) and the Y2 receptor antagonist BII0246 (0.03 mmol/kg) on visceral pain sensitivity were evaluated in conjunction with intrarectal administration of AITC (2%) to C57BL/6 N mice. The results revealed that the Y2 receptor antagonist increased pain-related behaviors induced by intrarectal AITC while the Y2 receptor agonist had no effect.

**Figure 6:**
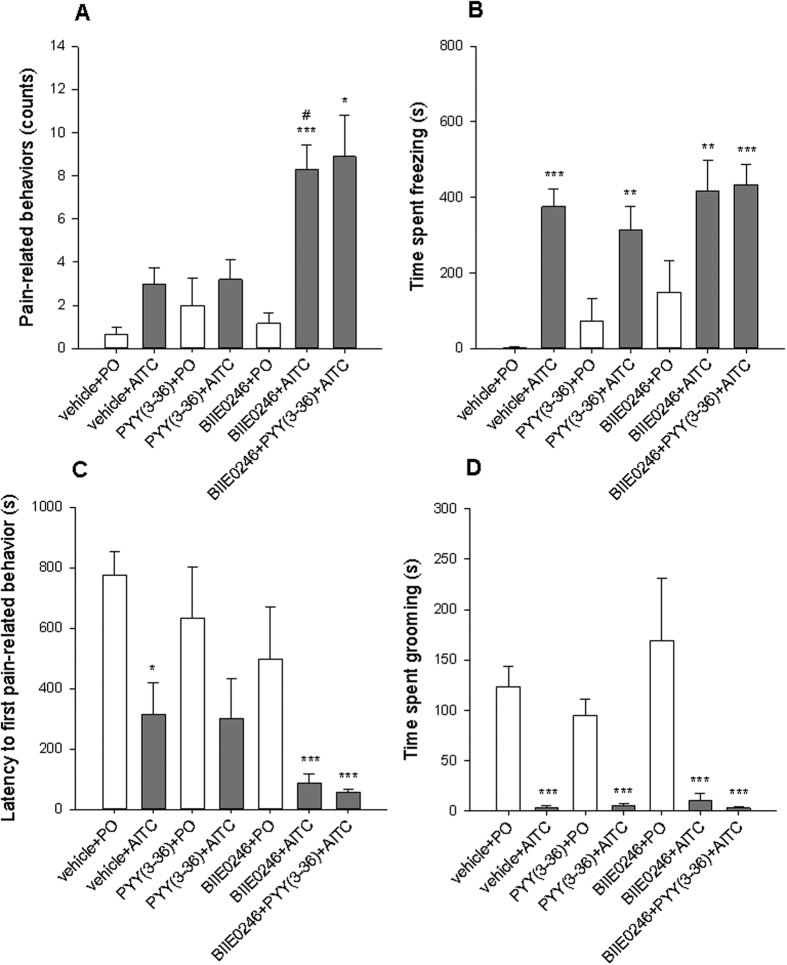
Effects of subcutaneously injected BIIE0246 (0.03 mmol/kg) and intraperitoneally injected PYY(3–36) (0.2 mg/kg) on pain evoked by intrarectally administered PO or AITC (2%, 0.05 ml) in C57BL/6 N mice. Shown are pain-related behaviors (**A**), time spent freezing (**B**), latency to first pain-related behavior (**C**), and time spent grooming (**D**). The behavioral parameters were evaluated blindly from 15-min videos recorded immediately after intrarectal treatment. One-way ANOVA with Welch’s correction revealed significant differences among the groups. The data shown are means + SEM, n = 6 for PO groups and 9–12 for AITC groups; *p < 0.05, **p < 0.01, ***p < 0.001 compared to vehicle + PO, ^#^*p* < 0.05 compared to vehicle + AITC.

As depicted in Fig. 6, one-way ANOVA with Welch’s correction disclosed statistically significant differences among the groups with regard to pain-related behaviors (Welch’s F_(6,21.5)_ = 9.4; *p* < 0.001), time spent freezing (Welch’s F_(6,19.1)_ = 26.1; *p* < 0.001), latency to first pain-related behavior (Welch’s F_(6,18.2)_ = 15.6; *p* < 0.001), and time spent grooming (Welch’s F_(6,18.7)_ = 10.9; *p* < 0.001).

Post-hoc testing failed to reveal any significant differences in pain-related behavior counts between the vehicle + PO group and vehicle + AITC group. Significantly higher pain-related behavior counts were recorded in the BIIE0246 + AITC and BIIE0246 + PYY(3–36) + AITC groups compared to the vehicle + PO and BIIE0246 + PO groups. The BIIE0246 + AITC group also exhibited significantly higher pain-related behavior counts than the vehicle + AITC group (Fig. 6A). The time spent freezing was significantly higher in all groups receiving AITC compared to PO, but no significant differences were observed within the groups receiving PO or within the groups receiving AITC (Fig. 6B). Similarly, the time spent grooming and the latency to the first pain-related behavior were shorter in all groups receiving AITC than in the PO group, while no significant differences were observed within groups receiving PO or within groups receiving AITC (Fig. 6C,D). The parameters recorded by the LabMaster system (horizontal activity, vertical activity, traveling distance) measured for 15 min after intrarectal injection of PO or AITC were not significantly different among the experimental groups (Supplement, Figure S3).

## Discussion

Although the role of Y receptors in nociception is well established^9^, relatively little is known about the specific implication of PYY in pain regulation. In this work, we examined the effects of genetic PYY deletion and Y2 receptor ligands on somatic sensitivity to thermal and mechanical pain and visceral sensitivity to chemical pain. To probe chemonociception in the rectum we used AITC which stimulates transient receptor potential ankyrin 1 (TRPA1) and transient receptor potential vanilloid 1 (TRPV1) ion channels expressed by nociceptive afferent nerve fibers^21^,^22^. On the somatic level, PYY^(−/−)^ mice were found to be hypersensitive to thermal somatic pain. On the visceral level, PYY knockout exaggerated pain-related behaviors in response to intrarectal AITC. In confirmation of these results, the Y2 receptor antagonist BII0246 increased pain-related behaviors in response to intrarectal AITC. These findings attest to an involvement of PYY in the regulation of somatic and visceral pain sensitivity.

To study visceral pain and hyperalgesia by a minimally invasive method that does not require surgery, we took advantage of the models of Laird *et al*.^18^ and Langford *et al*.^20^ and the recording of locomotor activity^19^ to gauge pain by a multitude of behavioral readouts considered to reflect several dimensions of pain experience. By recording several behavioral manifestations of visceral pain (stretching, squashing, arching, licking of the abdomen)^18^ we found that intrarectal AITC evoked pain-related behaviors only in PYY^(−/−)^ mice but not in WT mice. It appears as if PYY knockout enhances the susceptibility of the mice to respond to the stress and mechanical irritation associated with the instillation procedure with pain-related behaviors. This phenotype is consistent with impaired stress coping and enhanced depression-like behavior seen in PYY^(−/−)^ mice^23^. The moderate behavioral response of the WT mice to intrarectal AITC was untypical when compared with the more pronounced response observed in C57BL/6 mice^19^,^24^. Although the reason for this discrepancy is not understood, we think of a strain effect, given that the genetic background of the WT animals is different from that of C57BL/6 mice.

The MGS described by Langford *et al*.^20^ is a novel method to assess the pain experience in response to several noxious stimuli. In addition, recording of the MGS does not interfere with the evaluation of pain-related behaviors or referred hyperalgesia. On the basis of our study we contend that a combination of several behavioral methods to assess visceral pain, including facial pain expression, a variety of pain-related behaviors and referred hyperalgesia, addresses several dimensions of nociception and has the potential to overcome strain-related differences in the nociceptive responses to noxious stimuli. Although, for instance, the WT mice used here did not show the typical pain-related behaviors described by Laird *et al*.^18^ in response to intrarectal AITC, they exhibited a very prominent MGS response: the ΔMGS was more than 20 fold larger in response to AITC than after administration of PO. On the other hand, the effect of PYY knockout on MGS was not statistically significant.

A particular dimension of the visceral pain experience manifests itself in the phenomenon of referred pain. We used a decrease in the MPT of the hindpaws as an index of referred hyperalgesia and found that the AITC-induced reduction of the MPT was most pronounced in PYY^(−/−)^ mice. It must not be overlooked, however, that the AITC-induced ΔMPT in PYY^(−/−)^ mice was only nominally, but not significantly, different from the AITC-induced ΔMPT in WT mice. The absence of a significant AITC-evoked referred hyperalgesia in WT mice is consistent with the relative resistance of this genotype towards pain-related behaviors induced by AITC. It is worth noting, however, that in WT but not PYY^(−/−)^ mice administration of the vehicle (PO) increased the MPT of the hindpaws. This rise of the MPT may be interpreted as stress-induced analgesia which is known to be strain dependent^25^,^26^. If so, the MPT changes in the different experimental groups may represent the composite result of stress-induced analgesia and referred hyperalgesia evoked by intrarectal irritation.

Since PYY(3–36), the circulatory form of PYY, has a higher affinity for Y2 receptors than PYY itself^5^, we examined the effects of this Y2 receptor agonist (0.2 mg/kg) and the Y2 receptor antagonist BIIE0246 (0.03 mmol/kg) on pain-related behaviors induced by AITC (2%) in C57BL/6 N mice. The experiments revealed that Y2 receptor antagonism led to visceral hyperalgesia in response to intrarectal AITC, while Y2 receptor agonism had no effect. Specifically, PYY(3–36) failed to attenuate pain-related behaviors and to attenuate the effect of BII0246 to exaggerate AITC-induced pain-related behaviors. The inefficacy of PYY(3–36) could be due to pharmacodynamic reasons, pharmacokinetic factors and/or particular experimental conditions. First, the simplest explanation would be to assume that dosing of PYY(3–36) was inadequate. It should be noted, however, that the dose used here (0.2 mg/kg) is considered to be a high dose, given that doses as low as 0.01 mg/kg are sufficient to enhance c-Fos expression in the brain and to induce several behavioral changes in mice^27^,^28^. Second, the failure of PYY(3–36) to antagonize the hyperalgesic effect of BII0246 may be due to different pharmacokinetic profiles of the two compounds, PYY(3–36) being unable to achieve effective concentrations at the site where BII0246 exerts its hyperalgesic action. Third, it should not go unnoticed that a failure of PYY(3–36) to induce expected pharmacological effects is a common observation in the literature^29^. The inconsistent efficacy of PYY(3–36) *in vivo* has been attributed to the stress in handling the animals, its poor pharmacokinetic profile in rodents, or concomitant activation of Y1 and Y5 receptors, as PYY(3–36) is not totally receptor-selective^30^,^31^.

The findings of this study that PYY knockout and Y2 receptor antagonism enhance particular aspects of AITC-evoked visceral pain behavior raise the question as to the site in the pain pathway where PYY and Y2 receptors operate. Y1 and Y2 receptors are widely expressed in the primary somatic and visceral pain pathways, including the skin and colon, the inferior ganglion of the vagus nerve, the dorsal root ganglia, and the dorsal horn of the spinal cord^9^,^32^,^33^,^34^,^35^. An implication of the NPY system in AITC-evoked rectal pain can be deduced from the increased expression of NPY and Y1 receptor mRNA in the spinal cord of PYY^(−/−)^ mice. The functional implication of these findings is not clear. However, given that NPY and Y1 receptor signaling in the spinal cord have an analgesic function^8^, it could be contended that the increase in spinal NPY and Y1 receptor mRNA expression in PYY^(−/−)^ mice reflects a counterregulatory mechanism to balance the hyperalgesic state in this genotype. Due to the widespread distribution of multiple Y receptors in the peripheral and central pain pathways it is difficult to hypothesize on the sites where PYY could influence visceral and somatic pain, given that PYY can cross the blood brain barrier^36^. The observation that both somatic and visceral pain sensitivity was increased in PYY^(−/−)^ mice is compatible with both a peripheral and central site of action. In contrast, BII0246 acts primarily by blocking peripheral Y2 receptors^37^, which suggests that peripheral Y2 receptors play a role in controlling visceral pain signaling. This contention is in keeping with the expression of Y2 receptors by vagal and spinal primary sensory neurons in rodents and rabbits^9^,^32^,^38^.

In conclusion, PYY knockout and Y2 receptor antagonism have a hyperalgesic effect in mice, exaggerating particular behavioral aspects of both somatic and visceral pain. Circumstantial evidence indicates that the effect on visceral pain is mediated by peripheral Y2 receptors. Our experimental observations in mice may have a translational value in view of the deficiency of PYY reported to occur in the colon of IBS and IBD patients^12^,^13^,^14^,^15^. The results of the current work suggest that the decreased PYY content in the lower GIT of IBS and IBD patients may contribute to the visceral pain associated with these pathologies.

## Methods

### Experimental animals

PYY^(−/−)^ mice were generated by removing the entire coding sequence including the initiation start as reported previously^39^. The genetic background of the knockout as well as WT mice was a 1:1 mixture of C57Bl/6 and129/SvJ. The PYY^(−/−)^ mice and the age-matched WT mice were 2–4 months old when used in the plantar test and in the assessment of spontaneous pain-related behaviors and 3–9 months old when MGS and referred pain were assessed in parallel. Three different lots of mice were used for the plantar test, the assessment of spontaneous pain-related behaviors, and the combined assessment of MGS and referred pain. The animals subjected to the MGS and referred pain measurements were also used to assess the expression of NPY, Y1 receptor and Y2 receptor expression in the spinal cord. The total number of WT and PYY^(−/−)^ mice employed in this study amounted to 77 male mice.

The effects of BII0246 and PYY(3–36) on pain behavior were evaluated in a total of 59 male C57BL/6 N mice obtained from Charles River (Sulzfeld, Germany). The C57BL/6 N mice arrived in the institutional animal house at 8 weeks of age and were housed 2–3 per cage. They were habituated for 2 weeks before they were subjected to the experiments.

### Ethical statement

All experiments were approved by an ethical committee at the Federal Ministry of Science, Research, and Economy of the Republic of Austria (BMWFW-66.010/0054-WF/II/3b/2014) and conducted according to the Directive of the European Communities Council of 24 November 1986 (86/609/EEC) and the Directive of the European Parliament and of the Council of 22 September 2010 (2010/63/EU).

### Intrarectal administration

Chemically induced nociception in the colon was induced by intrarectal AITC administered through a Teflon feeding cannula (length 38.1 mm, gauge 20) (Scanbur, Karlslunde, Denmark). Vaseline (Rösch & Handel, Vienna, Austria) was applied to the perianal region and the tip of the cannula to reduce any discomfort induced by the insertion of the cannula. AITC (1% (v/v)) in a volume of 0.05 ml in the MGS and referred pain experiment, 2% in a volume of 0.1 ml in assessment of pain related behavior in WT and PYY^(−/−)^ mice and 2% in a volume of 0.05 ml in assessment of pain related behaviors in C57BL/6 N mice or its vehicle, PO, was injected intrarectally through the feeding cannula^18^,^19^,^21^. The concentration (1%) and volume (0.05 ml) of AITC used in the MGS and referred pain experiment was chosen to avoid any ceiling effect as noted with higher dosing in pilot trials.

### Drug treatment

PYY(3–36) lyophilized powder (Sigma-Aldrich, Vienna, Austria) was dissolved in saline (0.9% NaCl) containing 1% bovine serum albumin. Aliquots were stored at −70 °C in polypropylene tubes and thawed on the day of experiment. The peptide was injected intraperitoneally at the dose of 0.2 mg/kg (volume: 0.05 ml/10 g body weight). This dose of PYY(3–36) is sufficient to induce several behavioral changes in mice^27^. BIIE0246 (Tocris, Bristol, UK) was dissolved in 30% polyethylene glycol 200 in distilled water, prepared freshly on the day of the experiment and injected subcutaneously (SC) at the dose of 0.03 mmol/kg (volume: 0.05 ml/10 g body weight). This dose of BIIE0246 was used previously to evaluate the role of Y2 receptors in vagal afferent signaling^10^. To assess the effects of the Y2 receptor agonist PYY(3–36) and the Y2 receptor antagonist BIIE0246, 7 groups of mice were used. The time line of the experiments was such that, first, the mice received a SC injection of BIIE0246 or its vehicle, 5 min later IP PYY(3–36) or its vehicle, and after a further 5 min PO or 2% AITC intrarectally. The 7 experimental groups were as follows:

1. SC vehicle, IP vehicle, PO intrarectally (vehicle + PO)
2. SC vehicle, IP vehicle, 2% AITC intrarectally (vehicle + AITC)
3. SC vehicle, IP PYY(3–36), PO intrarectally (PYY(3–36) + PO)
4. SC vehicle, IP PYY(3–36), 2% AITC intrarectally (PYY(3–36) + AITC)
5. SC BIIE0246, IP vehicle, PO intrarectally (BIIE0246 + PO)
6. SC BIIE0246, IP vehicle, 2% AITC intrarectally (BIIE0246 + AITC)
7. SC BIIE0246, IP PYY(3–36), 2% AITC intrarectally (BIIE0246 + PYY(3–36) + AITC)

After intrarectal administration of 2% AITC, pain-related behaviors and locomotion were assessed as described below.

### Assessment of pain-related behaviors

After intrarectal administration of AITC or vehicle, the behavior of mice was video-recorded in the LabMaster system (TSE Systems, Bad Homburg, Germany) for 15 min. A blinded trained investigator used the event marker module of the Videomot 2 tracking software (TSE Systems) to evaluate the video-recordings with regard to the following pain-related behaviors^18^,^19^,^24^: (a) stretching of the trunk and squashing (pressing the abdomen towards the floor of the cage and stretching of the body), (b) licking of the lower part of the abdomen, and (c) arching of the trunk.

The time spent freezing and grooming was also calculated from the video-recordings. Horizontal and vertical locomotor activity was measured simultaneously with the LabMaster system^19^. Details about the LabMaster system are available in the supplement.

### The mouse grimace scale (MGS)

Facial pain expression was evaluated with the MGS as described previously^20^,^40^ with a slight modification that allowed for the simultaneous assessment of referred hyperalgesia. Mice were kept on a wire mesh (Ugo Basile) in a homemade plexiglass box (9 cm × 5 cm × 5 cm; length × height × width). All sides of the box except one (9 cm × 5 cm) were covered with white paper. Mice were left to habituate in the box for 30 min. Thereafter a 20-min video recording under baseline conditions was taken with a Canon Legira HF R406 video camera. After taking the video, MPT was measured as described later. Afterwards the mice received 1% AITC or the vehicle (PO) intrarectally as described above, followed by another 20-min video recording for the assessment of the MGS and a second measurement of MPT. The MGS was assessed as described in the supplement. The ΔMGS value was calculated by subtracting the baseline MGS from post-treatment MGS and used as an index of visceral pain. One hour after intrarectal treatment the mice were sacrificed by decapitation after they had been deeply anesthetized with pentobarbital (150 mg/kg IP). The lumbosacral spinal cords were collected, shock frozen in liquid nitrogen and stored at −70 °C until RNA extraction.

### Assessment of mechanical pain threshold (MPT) and referred pain

Referred pain^18^,^24^ was evaluated in parallel with the MGS assessment and determined by two measurements of MPT over the plantar surface of the hind paws and abdomen. The first measurement of MPT was made under baseline conditions after the baseline MGS video had been taken. The second measurement of MPT was taken 20 min after intrarectal administration of PO or 1% AITC when the video-recording of the post-treatment MGS had been completed. The MPT was evaluated with von Frey filaments (Bioseb, Vitrolles, France) using the simplified up-down method (SUDO method)^41^ as described in the supplement. The ΔMPT value was calculated by subtracting the baseline MPT from the post-treatment MPT. A reduction of ΔMPT was considered as an index of referred pain.

### Real time PCR

NPY, Y1 receptor and Y2 receptor mRNA expression in the lumbosacral spinal cord was assessed by real-time PCR. RNA was extracted with the RNeasy Lipid Tissue Mini Kit (Qiagen, Hilden, Germany). Reverse-transcription was performed with the High Capacity cDNA Reverse Transcription kit as described by the manufacturer (Applied Biosystems, Foster City, CA, USA). The PCR conditions and the primer sequences are described in the supplement. GAPDH and PGK were used as reference genes. Quantitative values of mRNA relative to control were calculated with the 2^−ΔΔCT^ method^42^.

### Statistical analysis

SPSS 22 and SigmaPlot 13 were used for statistical analysis and graphic presentation of the results. The data were analyzed with two-sample *t*-test, paired t-test, or one-way ANOVA, as appropriate. Post hoc pairwise comparisons with Tukey’s test were made when one-way ANOVA revealed significant differences among the groups. Log transformation was considered whenever needed to meet one-way ANOVA assumptions. When log transformation was not sufficient to meet the equal variability assumption of one-way ANOVA as tested by Levene’s test, the Welch correction and post-hoc Games-Howell test were used. Differences between groups in *t* test, one-way ANOVA, and post-hoc tests were considered significant if *p* ≤ 0.05. All data are presented as means + SEM, n referring to the number of mice in each group.

## Additional Information

**How to cite this article**: Hassan, A. M. *et al*. Visceral hyperalgesia caused by peptide YY deletion and Y2 receptor antagonism. *Sci. Rep.* **7**, 40968; doi: 10.1038/srep40968 (2017).

**Publisher's note:** Springer Nature remains neutral with regard to jurisdictional claims in published maps and institutional affiliations.

## Supplementary Material

Supplementary Figures and Methods
