# Supplementary material for: Visceral hyperalgesia caused by peptide YY deletion and Y2 receptor antagonism
Source: Sci Rep. 2017 Jan 20;7:40968. doi: 10.1038/srep40968 (PMC5247702; doi:10.1038/srep40968)
Supplement: Supplementary Figures and Methods [file srep40968-s1.doc]

# **Supplementary information**

# Visceral hyperalgesia caused by peptide YY deletion and Y2 receptor antagonism

Ahmed M Hassan^a§^, Piyush Jain^a§^, Raphaela Mayerhofer^a^, Esther E. Fröhlich^a^, Aitak Farzi^a^ , Florian Reichmann^a^, Herbert Herzog^b^, Peter Holzer^a^*

^a^Research Unit of Translational Neurogastroenterology, Institute of Experimental and Clinical Pharmacology, Medical University of Graz, Universitätsplatz 4, 8010 Graz, Austria

^b^Neurobiology Research Program, Garvan Institute of Medical Research, 384 Victoria Street, Darlinghurst, Sydney, NSW 2010, Australia

^§^Contributed equally

*Correspondence should be addressed to P.H. (email: [peter.holzer@medunigraz.at](mailto:peter.holzer@medunigraz.at))

# **Supplementary figures**


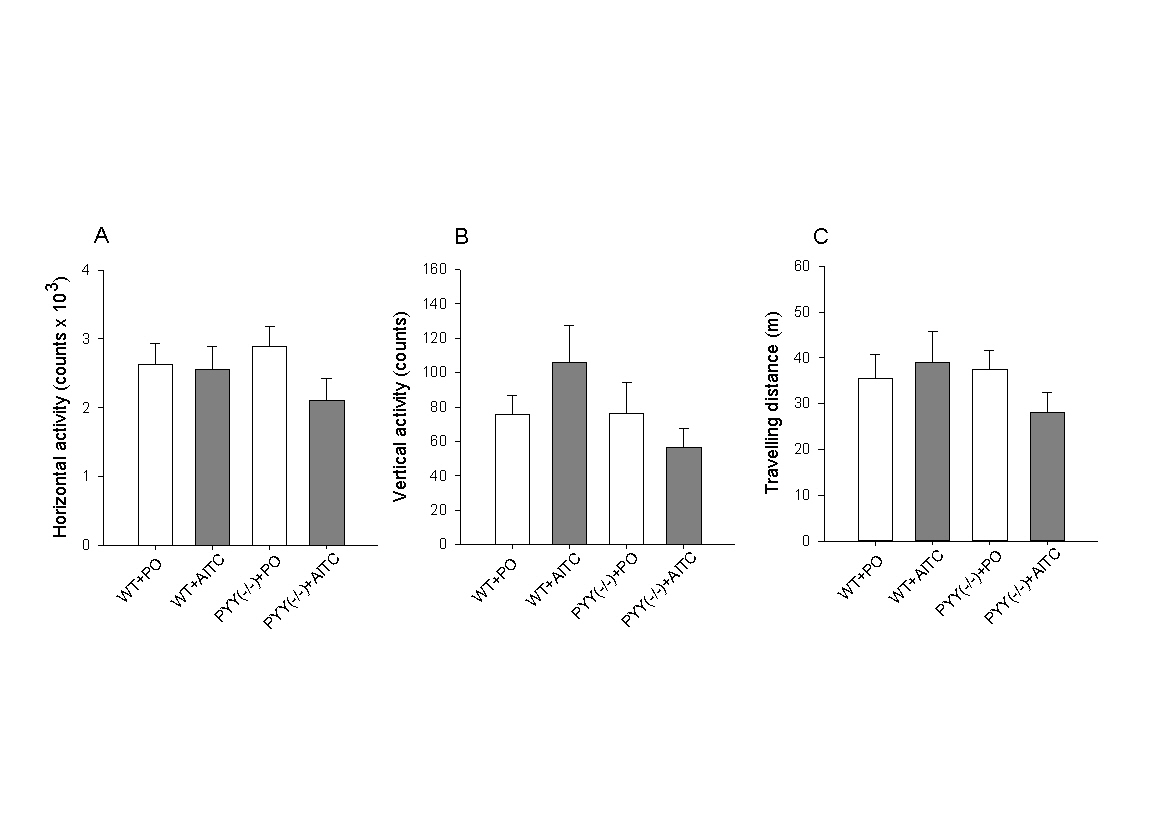


Figure S1: Effect of intrarectally administered AITC (2%, 0.1 ml) and PYY knockout (PYY (-/-)) on horizontal locomotor activity (A), vertical locomotor activity (B), and traveling distance (C). These parameters were recorded with the LabMaster system for a 15-min period immediately after intrarectal treatment. One-way ANOVA revealed no significant differences among the groups. The data shown are means + SEM, n=6-8 per group.


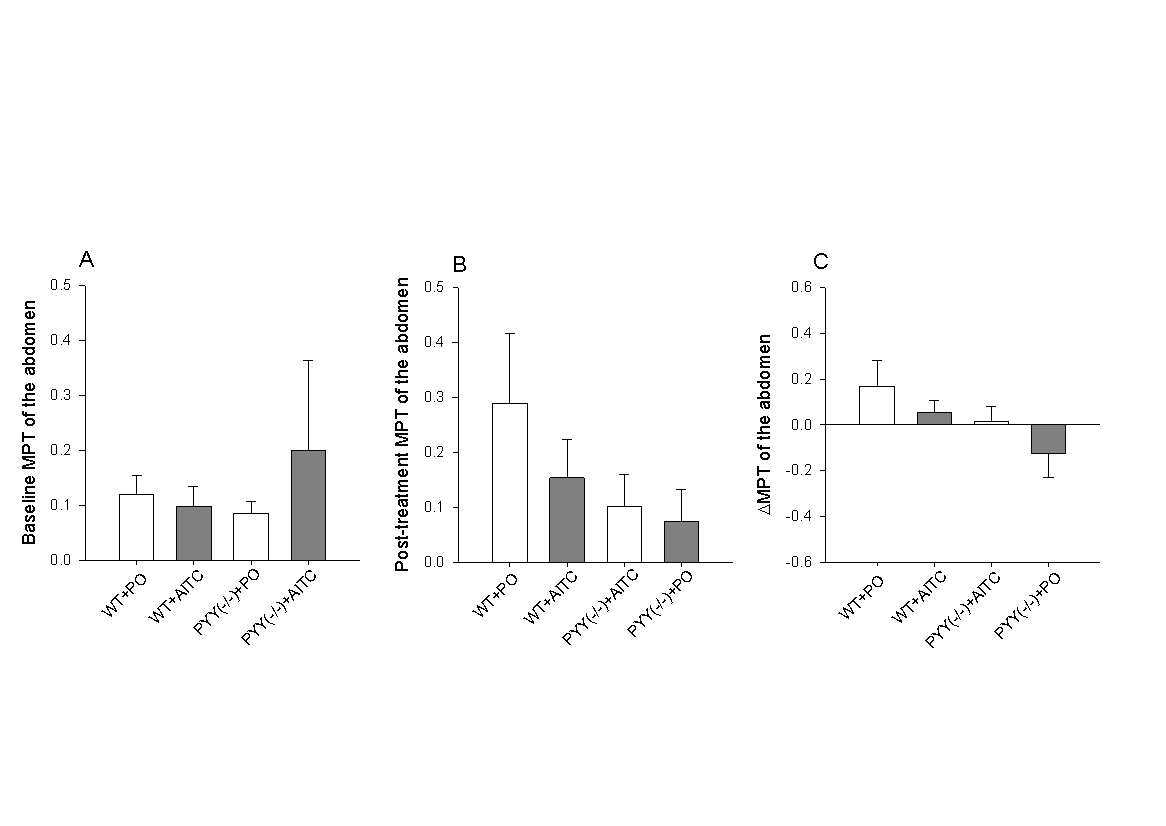


Figure S2: Mechanical pain threshold (MPT) of the abdomen in WT and PYY knockout (PYY (-/-)) mice before (baseline, A) and after (post-treatment, B) intrarectal administration of PO or AITC (1%, 0.05 ml) and the difference between the two measurements (ΔMPT) (C). MPT was assessed with von Frey hairs, and the values represent the average of two measurements. One-way ANOVA failed to disclose any significant differences among the groups. The data shown are means + SEM, n=5-7 per group.

**
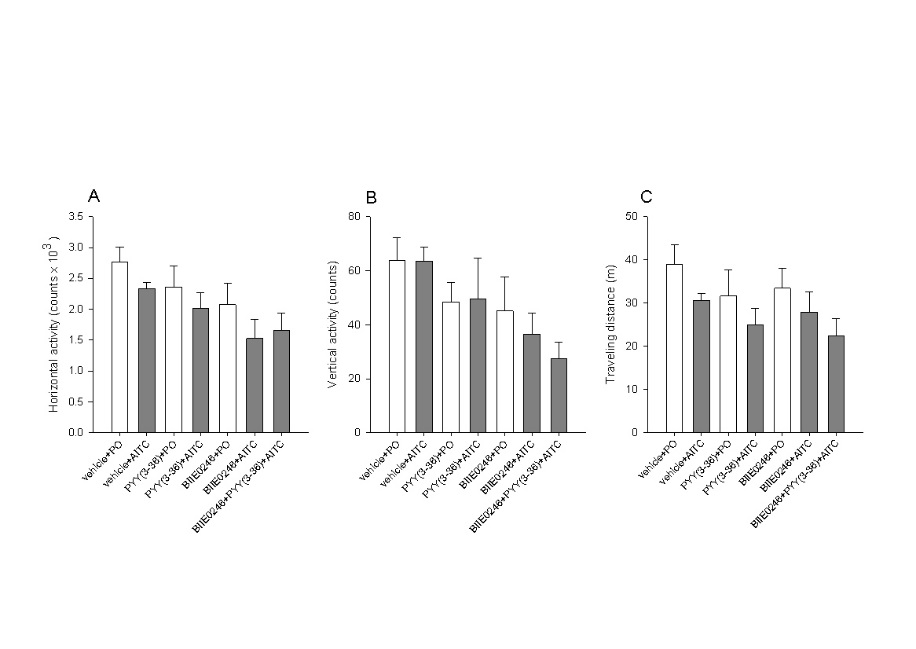
**

Figure S3: Effects of subcutaneously injected BIIE0246 (0.03 mmol/kg) and intraperitoneally injected PYY(3-36) (0.2 mg/kg) on horizontal locomotor activity (A), vertical locomotor activity (B), and traveling distance (C) recorded after intrarectal administration of PO or AITC (2%, 0.05 ml) in C57BL/6N mice. These parameters were evaluated with the LabMaster system for 15 min immediately after intrarectal treatment. One-way ANOVA revealed no significant differences among the groups. The data shown are means + SEM, n=5-6 for PO groups and 9-12 for AITC groups.

# **Supplementary methods**

## LabMaster recording of locomotor activity

The LabMaster system (TSE Systems, Bad Homburg, Germany) was used to assess locomotor activity. The system allows continuous recording of the animals without intervention by any investigator. As described previously^1, 2^, the LabMaster system consists of test cages (type III, 42.0 X 26.5 X 15.0 cm, length X width X height), surrounded by two external infrared frames and a cage lid equipped with three weight transducers (for assessment of ingestive behavior). For recording locomotion and exploration, the two external infrared frames are positioned in a horizontal manner above one another at a distance of 4.3 cm, with the lower frame being fixed 2.0 cm above the bedding floor. The bottom frame is used to record horizontal activity of the mice, whereas the top frame detects vertical movements (rearing, exploration). The measures of activity (locomotion, exploration) are derived from the light beam interruptions (counts) of the corresponding infrared frames.

Locomotor activity was measured for 15 min post-treatment in the absence of any food or fluid provided during this period. The cages were covered with pored transparent plates to allow video recording.

## Analysis of mouse grimace scale (MGS) videos^3^.

A blinded analyzer captured the frames that showed clear facial expressions from the video. The analyzer took the earliest frame showing a clear facial expression in the video as JPEG file, then started searching for another clear frame after 1.5 min. Since the mice were not always in a favorable position to take the next clear frame, the usual framing interval was 1.5-3 min.

The resultant JPEG files were cropped such that the body position was no longer visible. Then the following specific facial expression action units (AUs) were evaluated:

- - - 1. Orbital tightening which is a narrowing of the orbital area, with a tightly closed eyelid or an eye squeeze (denoted by a wrinkle around the eye).
      2. Nose bulge which is a rounded extension of the skin visible on the bridge of the nose.
      3. Cheek bulge which refers to a convex appearance of the cheek muscle (between eye and whiskers) relative to its baseline position, or shortening of the distance from eye to whisker pad.
      4. Position of the ears which can be rotated outward or back (away from the face) during pain.
      5. Position of the whiskers which can be pulled backward or forward, or be clumped together.

The score for each AU was 0 (not present), 1 (moderately visible) or 2 (clearly visible). Since the whiskers were not clear in many images, they were not included in the analysis, as MGS assessment without scoring of whisker position was reported previously^4^. After unblinding, all photos of each mouse under baseline and post-treatment conditions were compiled together, and the average baseline MGS and post-treatment MGS were calculated for each mouse.

## Measurement of mechanical pain threshold (MPT)

The MPT was evaluated with von Frey filaments (Bioseb, Vitrolles, France) using the simplified up-down method (SUDO method) ^5^. The forces applied with the test filaments were 0.02, 0.04, 0.07, 0.16, 0.4, 0.6, 1.0, and 1.4 g. Testing began with a filament of 0.16 g force for the paw and 0.07 g force for the abdomen. The testing sequence progressed with applications of 5 different force filaments following an up-down sequence such that a positive response to a filament indicated the next lower force filament to be used, while a negative response indicated the next higher force filament to be used. Finally, the MPT was calculated with the following formula: MPT = force of 5th hair applied + adjustment factor. The adjustment value equals + 0.5 of the last stimulus interval if the last hair produced a negative response. Alternatively, the adjustment factor equals - 0.5 of the last stimulus interval if the last hair produced a positive response. The MPT was measured on the plantar surface of both hindpaws while two consecutive measurements were taken over the abdomen. The hairs were applied for 1–2 s, with an inter-stimulus interval of at least 10 s. Over the belly, stimuli were applied on the lower to mid abdomen, avoiding the external genitalia, and care was taken not to stimulate the same point twice in succession. Withdrawal of the paw in response to hair application was rated as positive response to hindpaw stimulation, while sharp retraction of the abdomen, immediate licking or scratching of the site of hair application, and trials of escaping or jumping were considered positive responses to abdominal stimulation^6, 7^. The average MPT of both paws and of the two abdominal measurements were used in the statistics. ΔMPT was used as an index of a change in pain sensitivity and calculated by subtracting the baseline MPT from post-treatment MPT. Some mice showed prolonged squashing under pain conditions and did not respond to the hairs; these mice were excluded from the analysis.

## Real time PCR

For relative quantitation of mRNA, real time PCR was performed with the CFX Connect™ Real-Time PCR detection system in combination with the CFX Manager™ software 3.1 (Bio-Rad, Vienna, Austria). The specific primers used for amplification and quantitation of mRNA are presented in **Table S1**. Both GAPDH (Mm_Gapdh_3_SG QuantiTect Primer Assay, Qiagen, Hilden, Germany) and PGK were used as reference genes. The PCR SsoAdvanced™ Universal SYBR® Green Supermix (Bio-Rad, Vienna, Austria) was used for amplification, and the cycling conditions were as follows: samples were heated to 95 °C for 30 s followed by 39 cycles of 95 °C for 3 s, and 60 °C for 30 s. Except for the GAPDH primers which have been validated by the manufacturer, the products of all other primers were sequenced to confirm specificity. The sequencing was performed by VBC Biotech (Vienna, Austria). Quantitative values of mRNA relative to control were calculated with the 2^-ΔΔCT^ method^8^.

Table S1: Primers used in the study

| **Gene** | **Primer sequence (5' -->3')** | **Reference** |
| --- | --- | --- |
| **NPY forward** | CAGATACTACTCCGCTCTGCGACACTACAT | 9 |
| **NPY reverse** | TTCCTTCATTAAGAGGTCTGAAATCAGTGTCT |  |
| **PGK forward** | ATGTCGCTTTCCAACAAGCTG | Harvard PrimerBank^10^  ID: 6679291a1 |
| **PGK reverse** | GCTCCATTGTCCAAGCAGAAT |  |
| **Y1 receptor forward** | TGATCTCCACCTGCGTCAAC | Harvard PrimerBank  ID: 6754882a1 |
| **Y1 receptor reverse** | ATGGCTATGGTCTCGTAGTCAT |  |
| **Y2 receptor forward** | TCCGGGAATACTCCCTGATTG | Harvard  PrimerBank  ID: 48277108c2 |
| **Y2 receptor reverse** | GCAAAACGTACAGGATGAGCAG |  |

**NPY (neuropeptide Y), PGK (phosphoglycerate kinase 1)**

# **References**

1. Painsipp, E. *et al*. Neuropeptide Y and peptide YY protect from weight loss caused by Bacille Calmette-Guerin in mice. *Br. J. Pharmacol.* **170**, 1014-1026 (2013).

2. Farzi, A. *et al*. Synergistic effects of NOD1 or NOD2 and TLR4 activation on mouse sickness behavior in relation to immune and brain activity markers. *Brain Behav. Immun.* **44**, 106-120 (2015).

3. Langford, D. J. *et al*. Coding of facial expressions of pain in the laboratory mouse. *Nat. Methods* **7**, 447-449 (2010).

4. Leach, M. C. *et al*. The assessment of post-vasectomy pain in mice using behaviour and the Mouse Grimace Scale. *PLoS One* **7**, e35656 (2012).

5. Bonin, R. P., Bories, C. & De Koninck, Y. A simplified up-down method (SUDO) for measuring mechanical nociception in rodents using von Frey filaments. *Mol. Pain* **10**, 26-8069-10-26 (2014).

6. Laird, J. M., Martinez-Caro, L., Garcia-Nicas, E. & Cervero, F. A new model of visceral pain and referred hyperalgesia in the mouse. *Pain* **92**, 335-342 (2001).

7. Eijkelkamp, N. *et al*. Increased visceral sensitivity to capsaicin after DSS-induced colitis in mice: spinal cord c-Fos expression and behavior. *Am. J. Physiol. Gastrointest. Liver Physiol.* **293**, G749-57 (2007).

8. Schmittgen, T. D. & Livak, K. J. Analyzing real-time PCR data by the comparative CT method. *Nature protocols* **3**, 1101-1108 (2008).

9. Ferenczi, S., Zelei, E., Pinter, B., Szoke, Z. & Kovacs, K. J. Differential regulation of hypothalamic neuropeptide Y hnRNA and mRNA during psychological stress and insulin-induced hypoglycemia. *Mol. Cell. Endocrinol.* **321**, 138-145 (2010).

10. Spandidos, A., Wang, X., Wang, H. & Seed, B. PrimerBank: a resource of human and mouse PCR primer pairs for gene expression detection and quantification. *Nucleic Acids Res.* **38**, D792-9 (2010).
